# A Rasch analysis of the Burnout Assessment Tool (BAT)

**DOI:** 10.1371/journal.pone.0242241

**Published:** 2020-11-24

**Authors:** Emina Hadžibajramović, Wilmar Schaufeli, Hans De Witte

**Affiliations:** 1 Institute of Stress Medicine, Region Västra Götaland, Gothenburg, Sweden; 2 Biostatistics, Department of Public Health and Community Medicine, Institute of Medicine, University of Gothenburg, Gothenburg, Sweden; 3 Department of Psychology, Utrecht University, Utrecht, The Netherlands; 4 Research Unit Occupational & Organizational Psychology and Professional Learning, KU Leuven, Leuven, Belgium; 5 Optentia Research Focus Area, North-West University, Potchefstroom, South Africa; Medical University Innsbruck, AUSTRIA

## Abstract

Burnout as a concept indicative of a work-related state of mental exhaustion is recognized around the globe. Numerous studies showed that burnout has negative consequences for both individuals and organizations but also for society at large, especially in welfare states where sickness absence and work incapacitation are covered by social funds. This underlines the importance of a valid and reliable tool that can be used to assess employee burnout levels. Although the Maslach Burnout Inventory is by far the most frequently used questionnaire for assessing burnout, it is associated with several shortcomings and has been criticized on theoretical as well as empirical grounds. Thus, there is a need for an alternative questionnaire with a strong conceptual basis and proper psychometric qualities. This challenge has been taken up by introducing the Burnout Assessment Tool (BAT), according to which burnout is conceived as a work-related state of exhaustion among employees, characterized by extreme tiredness, reduced ability to regulate cognitive and emotional processes, and mental distancing. Given that the BAT is a new measure of burnout, its psychometric properties need to be evaluated. This paper focuses on an evaluation of the internal construct validity of the BAT using Rasch analysis in two random samples (n = 800, each) drawn from larger representative samples of the working population of the Netherlands and Flanders (Belgium). The BAT has sound psychometric properties and fulfils the measurement criteria according to the Rasch model. The BAT score reflects the scoring structure indicated by the developers of the scale and the BAT’s four subscales can be summarized into a single burnout score. The BAT score also works invariantly for women and men, younger and older respondents, and across both countries. Hence, the BAT can be used in organizations for screening and identifying employees who are at risk of burnout.

## Introduction

Burnout—a metaphor referring to a work-related state of mental exhaustion—was first used in the Unites States at the end of the 1970s [1]. Meanwhile the concept has spread around the globe and according to PsycINFO, the largest database of psychological research, over 12,000 peer reviewed scientific publications on the subject have appeared. Numerous studies showed that burnout has negative consequences both for individual employees as well as the organizations for which they are working. For instance, burnout is associated with poor physical and mental health of employees, such as type-2 diabetes, cardio-vascular disease, anxiety and depression [2]. In addition it leads to high replacement costs due to turnover and sickness absence [3] and work incapacitation [4], and to poor business outcomes in terms of job performance [5], safety [6], productivity [7] and quality of care [8]. Moreover, burnout is not only an individual and organizational problem, but also a problem for society at large, especially in welfare states where sickness absence and work incapacitation are covered by social funds. Hence it is not surprising that European legislation calls on employers to periodically assess psychosocial risks among their employees and to implement policies to prevent burnout and work stress. In a number of European countries, including Belgium and the Netherlands, burnout is recognized as an occupational disease or work-related disorder [9]. This underlines the importance of a valid and reliable tool that can be used to assess employee burnout levels.

Arguably, the gold standard for assessing burnout is the Maslach Burnout Inventory [10]. The MBI is based on the definition of burnout by Maslach and Jackson [11] as a syndrome of emotional exhaustion, depersonalization and reduced personal accomplishment, later referred to as exhaustion, cynicism and lack of professional efficacy, respectively [10]. Boudreau and Mauthe-Kaddoura [12] estimated that the MBI is used in 88% of all empirical studies on burnout. As a matter of fact, that means that burnout is what the MBI measures, and vice versa. This circularity and mutual dependence of concept and assessment—linked to the dominance of the MBI—is undesirable because it impedes new and innovative research that leads to a better understanding of burnout. Moreover, the MBI has been criticized on theoretical as well as empirical grounds. For instance, Schaufeli and Taris [13] have argued that rather than a constituting element of burnout, reduced professional efficacy should be considered as a consequence. Furthermore, it is maintained that reduced cognitive functioning was wrongfully excluded as a constituting element of burnout [14]. On the technical side, the MBI has been criticized for (a) skewed answering patterns that may affect its reliability [15]; (b) including positive (professional efficacy) items to assess a negative state [16]; (c) lack of clinically validated cut-off values [17]; (d) lack of statistical norms that are based on national representative samples [18] and (e) the fact that it yields three different subscale scores instead of a single burnout score [19].

Taken together, these criticisms call for an alternative self-report burnout instrument with a strong conceptual basis and proper technical qualities. This challenge has been taken up by introducing the Burnout Assessment Tool [20, 21]. The conceptual basis of the BAT builds on the analysis of Schaufeli and Taris [13], who argued that burnout represents both the inability and the unwillingness to expend effort, which is reflected by its energetic and motivational component, respectively. The unwillingness to perform manifests itself by increased resistance, reduced commitment, lack of interest, disengagement, and so on—in short, mental distancing. Thus, according to Schaufeli and Taris [13], inability (exhaustion) and unwillingness (distancing) are the key components that constitute two sides of the same burnout coin. The BAT was developed using a combination of an inductive and deductive approach. More specifically, 49 in-depth structured interviews were conducted with Flemish and Dutch professionals who frequently deal with persons who suffer from burnout, such as general practitioners, occupational physicians, occupational health psychologists, and career counselors. The aim was to find out which symptoms they would identify as being typical for burnout. For classifying the burnout symptoms mentioned in the interviews, the conceptual approach of Schaufeli and Taris [13] was used. Attention was also given to non-specific, atypical symptoms that are observed in other psychological disorders as well as in cancer, hypo- or hyperthyroidism, mood disorder or anxiety disorder.

As a final result, four symptom clusters emerged (see also the BAT test manual: www.burnoutassessmenttool.be) [21]. Not surprisingly, fatigue was mentioned unanimously as most important and distinctive for burnout (e.g., "exhaustion", "feeling empty", "completely exhausted", "having no energy", and "looking tired"). In addition, symptoms emerged that refer to cognitive and emotional impairment. Examples of the former are: "concentration problems", "making mistakes", "disturbed imprinting", "being less efficient", and "forgetfulness"; and of the latter: "weeping”, "irritability", "anger", "hot temper", and "being emotional". Finally, symptoms of mental distance were mentioned, such as "no motivation", "withdrawal", "finding one’s job meaningless", "indifference", and "cynicism".

Accordingly, burnout was described as a work-related state of exhaustion that occurs among employees, characterized by extreme tiredness, reduced ability to regulate cognitive and emotional processes, and mental distancing. Because of the exhaustion experienced, the necessary energy is lacking to adequately regulate one’s emotional and cognitive processes. In other words, when experiencing burnout, the functional capacity for regulating emotional and cognitive processes is impaired. This is subjectively experienced as a loss of emotional and cognitive control. By way of self-protection and in order to prevent further energy depletion and loss of control, mental distancing occurs. In this conceptualization, both sides of the burnout coin are represented by exhaustion and its concurrent cognitive and emotional impairment on the one hand, and mental distancing on the other hand. Based on this conceptualization of burnout, BAT items were carefully formulated and tested for four burnout symptoms: exhaustion, mental distance, and emotional and cognitive impairment (for further details see below). In addition to the four core symptoms, burnout is associated with secondary symptoms—psychological distress, psychosomatic complaints and depressed mood. These symptoms often occur together with burnout but are not specific to burnout.

Given that the BAT is a new measure of burnout, its psychometric properties need to be evaluated. Moreover, it needs to be tested whether the BAT can be used to obtain a single burnout score, which is impossible with the MBI. In a recently published paper, the measurement invariance of the BAT across seven cross-national representative samples was investigated, and the BAT was successfully modelled as a second-order factor with a good fit to the data [22]. The current paper focuses on an evaluation of the internal construct validity of the BAT using Rasch analysis. The Rasch measurement model, usually referred to as Rasch analysis, belongs to the modern psychometric approaches or item response theory (IRT). The Rasch model has been used in a variety of applications since its introduction in education during the 1950s, and has been widely used in the health sciences over the last two decades. Short introductions to Rasch analysis are described elsewhere [23–25] and a comprehensive overview of the statistical theory of Rasch models is given in a recent textbook [26]. The advantage of the Rasch model over the classical test theory approaches such as factor analysis is the lack of requirement for the normal score distribution. Hence it is the preferred choice for the analysis of ordinal data produced by multi-item questionnaires with ordered categorical responses. In addition, more detailed information about the persons, items and response categories is obtained in a more feasible way, as the Rasch analysis allows a unified approach to measurement issues such as unidimensionality, appropriate category ordering of polytomous items, testing the invariance of items, and differential item functioning (DIF) (these concepts are briefly explained in the method section below).

The Rasch measurement model [27] operationalizes the axioms of additive conjoint measurements, which are the requirements for the measurement construction [28–31]. In other words, the Rasch model is a mathematical model describing how data are expected to behave in order to approximate a unidimensional measurement with interval scale properties. A unique feature in Rasch analysis is that fitting the data to the Rasch model places both item and person estimates on the same log-odds units (logit) scale, and in the case of model fit these are independent parameters. Given that the data fit the Rasch model, construct validity and objective measurement is achieved and the total score is a sufficient statistic [26]. In case that the data do not fit the Rasch model, this is interpreted as an indication that the questionnaire does not have the right psychometric properties and hence needs to be revised and improved.

For instance, Rasch analysis of the MBI Student Survey (MBI-SS) among US preclinical medical students showed that the three MBI scales function adequately but not optimally, so the authors recommend including additional items and increasing the number of response options from seven to nine [32]. Another study examined the MBI Human Service Surveys (MBI-HSS) among Dutch nursing graduates and found problems with disordered response ordering for all items, as well as redundancy of the personal accomplishment scale [33]. Finally, a study among UK pediatric oncology staff showed that emotional exhaustion and personal accomplishment seem to work well, but that the depersonalization subscale is problematic [34]. Hence, it appears that occasional Rasch studies with the MBI show mixed results, suggesting that MBI data do not fit the Rasch model unequivocally. Besides, these studies used specific occupational and student samples, so results cannot be generalized beyond these groups.

The current paper reports on a Rasch analysis of the BAT using two representative samples of the working population of the Netherlands and Flanders (Belgium), respectively. More specifically, the aims of this study are to evaluate: (a) the BAT’s construct validity using Rasch analysis; (b) whether the BAT’s four subscales can be combined into a single burnout score; (c) possible differential item functioning regarding gender, age and country.

## Material and methods

### Sample

Data come from two representative samples of national working populations in terms of age, gender and industry in the Netherlands (n = 1500) and Flanders (n = 1500), collected in the summer of 2017. Details about the sampling procedure and sample characteristics are described in the BAT test manual [21]. The study was reviewed and approved by the Social and Societal Ethics Committee (SMEC) of KU Leuven (https://www.kuleuven.be/english/research/ethics/committees/smec) on October 22, 2015 (reference number: G-2015 10 353). Before filling out the questionnaire, participants were informed about the purpose of the study, that participation was voluntary, that they could stop at any moment if they wished to do so, that questions could be directed to a contact person (name and email address provided) and that complaints could be filed with the ethical committee (email address provided). Participants declared that they agreed with these terms by clicking on “next”. This consent procedure was approved by the ethical committee.

This study only considered complete cases for the analyses. Complete cases on all items were obtained for n = 2978 (NL = 1500, FL = 1478). Given the large sample sizes it was possible to use cross-validation to check the robustness of the results. Also, equal sizes for each of the compared groups are recommended for the evaluation of differential item functioning (DIF, explained below), to ensure that in case of DIF the largest group does not dominate the estimation of parameters [35]. Differential item functioning was evaluated for country (NL/FL), gender (male/female), and age (under/above the median age of 41). Therefore, the total sample was divided into four homogenous strata of men/NL, men/FL, women/NL and women/FL. Next a random sample of 200 respondents from each stratum was drawn twice, resulting in two subsamples of 800 individuals each (Table 1). The median age in the two samples was 41.

**Table 1:** Random samples used in Rasch analysis drawn from the representative samples of the working population of the Netherlands (NL) and Flanders (FL); count within each group.

	Sample 1 n = 800	Sample 2 n = 800
**Women/NL**	200	200
**Men/NL**	200	200
**Women/FL**	200	200
**Men/FL**	200	200
**≤41 years**	391	404
**>41 years**	409	396

### Measure

The Burnout Assessment Tool (BAT) is a self-report questionnaire consisting of 23 items (see S1 Appendix) grouped in four subscales: exhaustion (8 items), mental distance (5 items), cognitive impairment (5 items), and emotional impairment (5 items). All items are expressed as statements with five frequency-based response categories (1 = never, 2 = rarely, 3 = sometimes, 4 = often, 5 = always). The total burnout score is calculated as a mean of all 23 items, and a high score is indicative of high levels of burnout (range 1–5). The BAT also contains two subscales for secondary symptoms of psychosocial distress and psychosomatic complaints (five items each, not analyzed in this study). Detailed information about development of the BAT is described in the BAT test manual [20, 21].

#### The Rasch model

The goal of the Rasch analysis is to evaluate whether the observed data satisfy the assumptions of the Rasch model, in which case the measurement is construct valid. Important concepts in Rasch analysis are unidimensionality, monotonicity, invariance, DIF and local dependency.

*Unidimensionality* is a basic prerequisite for combining a set of items into a single burnout score, i.e. all items should represent a common latent trait. *Monotonicity* implies that the item responses are positively related to the latent trait. The response structure required by the Rasch model is a stochastically consistent item order; i.e. a probabilistic Guttman pattern [36]. This implies that persons experiencing higher levels of burnout are expected to get higher scores on the BAT and vice versa. Moreover, this pattern of responses needs to be observed across all response categories for each item. Analogously, increasing levels of severity of burnout across response categories for each item need to be reflected in the data. The *invariance* criterion implies that the items need to work invariantly across the whole burnout continuum for all individuals, i.e. the ratio between the location values (items’ positioning on the latent burnout logit scale) of any two items must be constant along the latent construct. Invariance also implies that the items need to work in the same way (invariantly) for all comparable groups, which is known as lack of *DIF*. The Rasch model contains only the latent variable and the items, and it is implicitly assumed that the model applies to all persons within a specific population. Thus, if a specific population contains both women and men, it is assumed that both the measurement model and the item parameters are the same for both groups. Simply put, given the same level of burnout, the scale should function in a similar way for both women and men.

*Local dependency* implies that, having extracted the unidimensional latent trait of burnout, there should be no other meaningful patterns in the residuals [23]. Local dependency may be violated by response dependency and/or multidimensionality [37] and has an effect on the fit of the data to the model. Response dependency can result in increased similarity of the responses of persons across items, so that responses are more Guttman-like than they should be under no dependency. Contrarily, multidimensionality results would result in responses being less Guttman-like than they should be under no dependency [38].

Response dependency occurs when items are linked in some way so that the response to one item depends on the response to another item. A known example from rheumatology is when several items assessing walking ability are included in the same questionnaire. If a person is able to walk several miles without difficulty, then that person is also able to walk 1 mile or less without difficulty [23]. In this way, items are response-dependent as there is no other logical way in answering the two items. Another form of response dependency, known as redundancy dependency, may be caused by the degree of overlap of the content of two items, so that a particular rating for one item implies logically the same rating for another item, e.g. two items reflecting reversed statements such as “I feel tired” and “I feel alert” [39]. Logically, multidimensionality occurs when items are measuring more than one latent dimension.

### Data analysis

Rasch analysis was performed on the two samples separately. A sample size of 800 is sufficient to yield a high degree of precision [40]. All analyses were done in RUMM2030 [41], where pairwise conditional maximum likelihood is used for computation of expected value estimates, based on the total raw scores (mean values of the 23 BAT items) and the actual response frequencies on each item, under the assumption that these observed scores fit the Rasch measurement model. Residuals, i.e. the differences between the model-expected values and the observed values, are scrutinized in several ways in order to evaluate whether the data fit to the Rasch model, where both items and individuals can be ordered according to their burnout levels on a common logit (burnout) scale. The partial credit model was used, which allows the distances between thresholds to vary across items [42]. To control for the large number of comparisons, the significance level was set at 0.01 and Bonferroni adjusted.

The first step was to investigate the BAT’s construct validity: We fitted the Rasch model to all 23 items and evaluated whether the items within each subscale would cluster together in a residual correlation matrix in a pattern that is consistent with the underlying conceptualization of the BAT. When instruments consist of a bundle of items measuring different aspects of the latent trait, it is expected that the correlation matrix of residuals reveals the clustering of the items within each subscale [39]. Any residual correlation between the items 0.2 above the average observed correlation is indicative of local dependency [39]. Moreover, in this step, the functioning of each item is evaluated in terms of (a) threshold ordering (i.e. appropriateness of the response categories, evaluated graphically and by thresholds estimates for each item); (b) discriminant ability (item fit residual within range of ± 2.5); (c) the non-significant item χ^2^ statistic; (d) local dependency (residual correlation matrix), and (e) absence of DIF for age, gender and country.

The assumption of unidimensionality was tested by Smith’s test of unidimensionality [43]. For this test, first a principal component analysis (PCA) on residuals was performed. Next, items loading positively and negatively on the first principal component were used to obtain an independent person estimate. In the next step, independent t-tests for differences in these estimates for each person were performed [43]. Less than 5% of such tests being outside the range of ±1.96 support the unidimensionality of the scale. A 95% binomial confidence interval of proportions [44] was used to show whether the lower limit of the observed proportion is below the 5% level [43]. When local dependency was detected we followed the method of combining correlated items into testlets, as recommended by Marais and colleagues [37, 38, 45]. This method combines correlated items into one or more testlets (preferably based on theoretical considerations) and the data are re-analyzed using testlets instead of individual items. Thus, the second step was to fit the model with the four testlets based on the BATs four subscales. The testlets’ model fit was compared with the fit obtained from the initial analysis of the individual items. The latent correlation among the subscales was also calculated, as well as the proportion of the non-error common variance accounted for when the testlets were added together to make a total score (also known as explained common variance) [45–47].

DIF was tested by conducting ANOVA of standardized residuals, which enables separate estimations of misfit along the latent trait, uniform and non-uniform DIF. It is important to distinguish between real and artificial DIF. As explained by Andrich and Hagquist [35, 48], artificial DIF is an artefact of the procedure for identifying DIF. Therefore, following their recommendation, DIF items detected by ANOVA were resolved sequentially; initial and resolved analyses were compared, and magnitude and impact of DIF were investigated [48, 49]. Real DIF can be dealt with by splitting a mis-fitting item into two items, e.g. one item for women, with missing values for men, and the other for men, with missing values for women, and subsequently reanalyzing the data. In addition to formal tests, DIF was also evaluated graphically by means of the item characteristic curve.

The adequacy of the fit to the Rasch model was evaluated by means of three overall summary fit statistics. The *item-trait interaction statistic* was computed to test whether the hierarchical ordering of the items was invariant across the burnout trait. A non-significant value of this χ^2^ statistic indicates invariance. Two other indices of the overall fit to the model are the *mean and standard deviations of items* and *persons residuals*. These were computed and compared to the model-expected values of a mean of zero and a SD of 1.

The *internal consistency* of the scale and the power of the BAT scale to discriminate among respondents with different levels of burnout were evaluated with the Person Separation Index (PSI). The PSI ranges from 0 to 1 and is similar to Cronbach’s alpha.

Targeting (distribution on a logit scale) of the BAT items and persons in the sample was evaluated graphically in a person-item-threshold graph. Targeting is an aspect of how well the items are targeted for severity levels of burnout as reported by the respondents. In a person-item-threshold graph the distribution of the person parameter estimates are compared with the distribution of the item thresholds. In that way, thresholds which are extreme compared to persons can be identified, as they provide little information in the population. This is important for the precision of person parameter estimates. In other words, responses to such items will have little impact on the precision of the person estimates as these items are out of target. For a well-targeted instrument, the mean location for persons would be around the value of zero.

Finally, in case of good fit to the model, Rasch person estimates, which are logits, can be transformed into a convenient range (henceforth referred to as metric score) [50].

## Results

### Rasch analysis on sample 1

In the first step, the Rasch model was fitted to all 23 items. The residual correlation matrix between the items is found in S2 Appendix in Table A1. Observed residual correlations indicated violation of local dependency. As expected, correlations higher than expected under the condition of local independence (in our sample a value >0.16) were found for most of the item pairs within each subscale and none between different subscales; exhaustion: EX1-EX4, EX1-EX8, EX3-EX4, EX3-EX5, EX3-EX8, EX4-EX7, EX4-EX8, EX7-EX8; mental distance: MD1-MD3, MD1-MD4, MD1-MD5, MD2-MD3, MD2-MD4, MD3-MD4, MD4-MD5; cognitive impairment: all pairs; and emotional impairment: EI1-EI2, EI1-EI4, EI1-EI5, EI2-EI4, EI2-EI5, EI3-EI4, EI3-EI5, EI4-EI5. The Smith’s test confirmed the presence of multidimensionality as the percentage of significant t-tests was 20.9 (CI 18.2;23.9) and thus confirmed the patterns observed in the correlation matrix (see Table 3, BAT 23 items). Overall fit statistics are presented in Table 3. The analysis on all 23 BAT items indicated poor fit to the model, with a significant χ^2^ statistic, and high standard deviation for mean person and item fit residuals.

**Fig 1:**
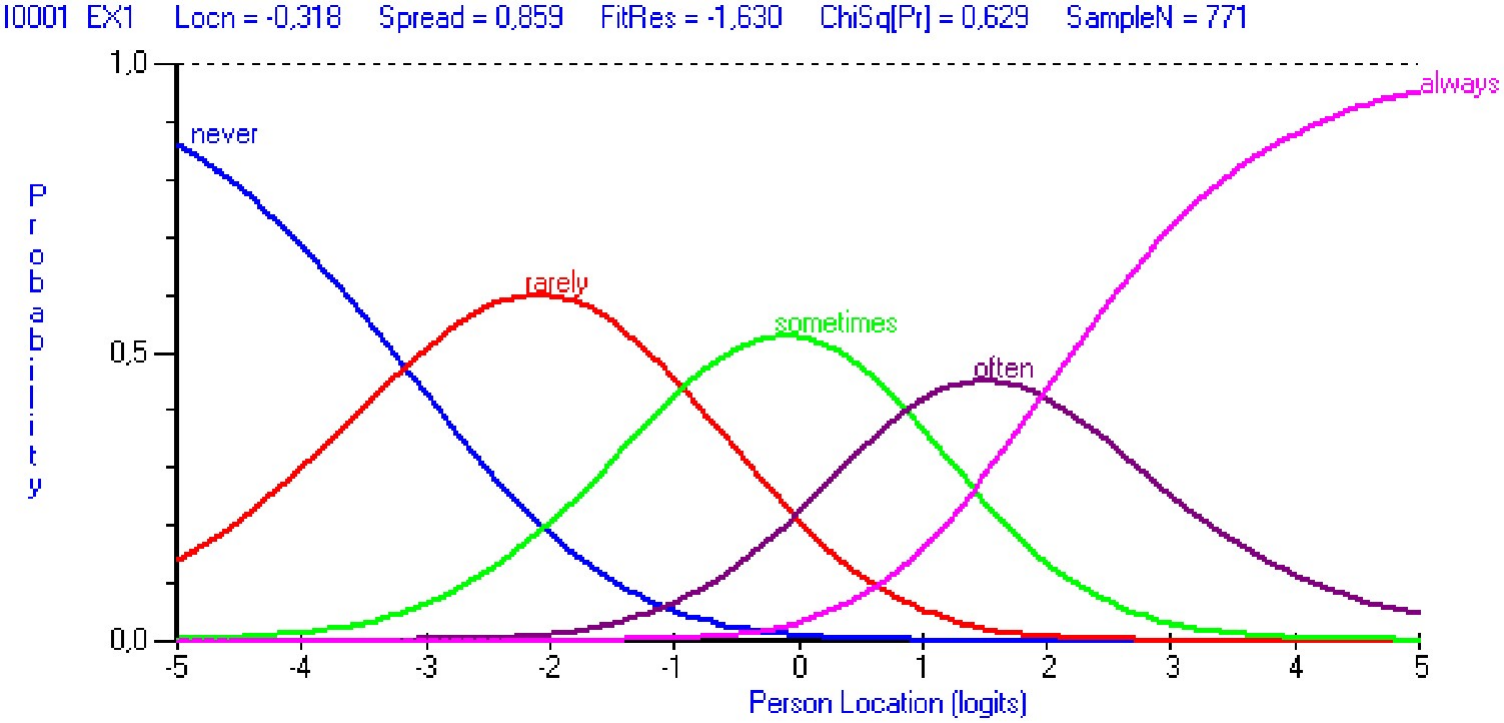
Category probability curves for the item EX1 (“At work, I feel mentally exhausted”).

Analyses on item level showed that all items had ordered thresholds. As seen in Fig 1, displayed as an illustrative example for item EX1, the probability of the response category *never* was highest at the lowest level of the latent estimate of burnout (person locations) and decreases when moving along the logit scale. In a similar way, the probability of choosing response categories implying higher levels of burnout increased with increasing levels of latent estimates of burnout.

**Fig 2:**
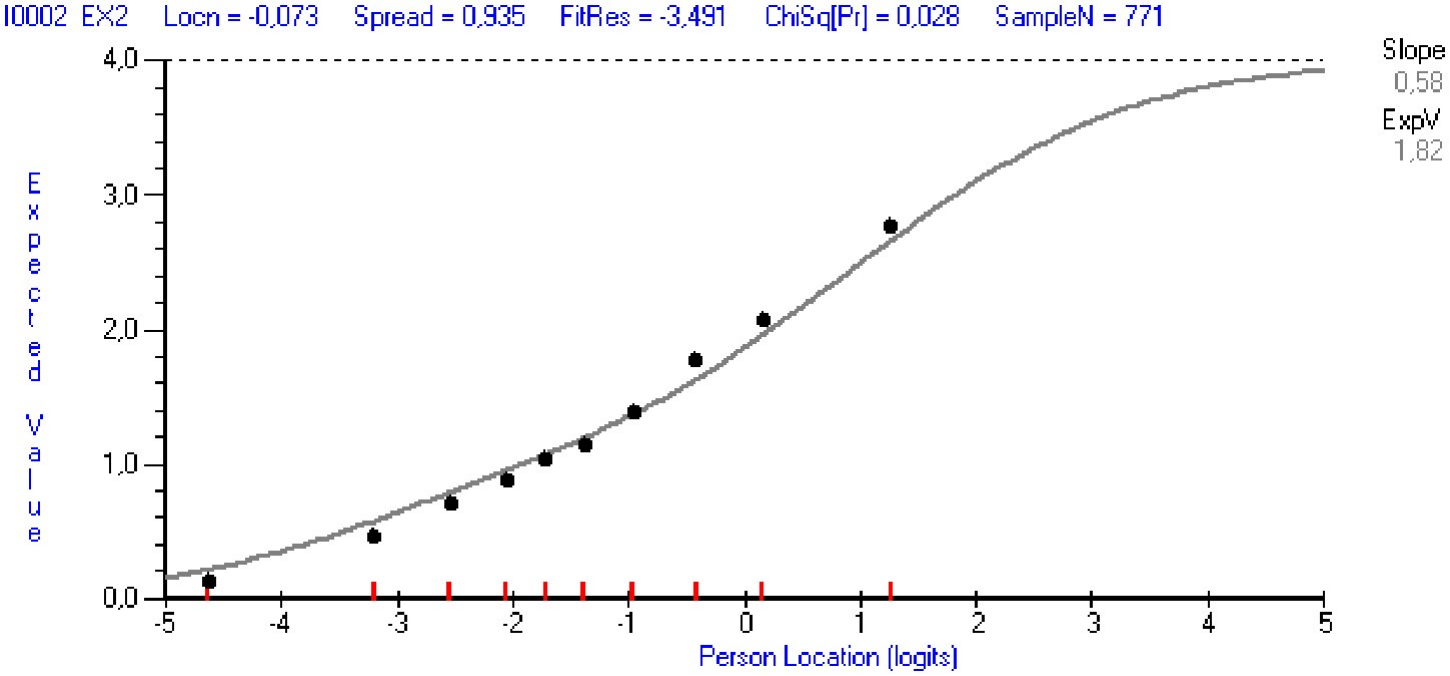
The item characteristic curve for EX2 (“Everything I do at work requires a great deal of effort”).

Item fit residuals outside the predefined range of ±2.5 were observed for exhaustion items EX2, EX7 and EX8, mental distance items MD1, MD2 and MD3, cognitive impairment item CI2 and emotional impairment items EI3, E4 and EI5 (Table 2). Among those, only items EX7 and MD2 showed a significant χ^2^ statistic. High positive and negative fit residual values are indicative of under- and over-discrimination of items respectively. However, visual examination of the item characteristic curves (ICC) showed that the observed values in most cases were located close to the expected value, as shown in Fig 2 for item EX2 as an illustrative example (the solid line represents expected values and dots are observed values within different class intervals). Table 2 shows item locations (i.e. the mean of threshold estimates), with a higher item location representing more severe burnout symptoms.

**Table 2:** Item locations, and fit residuals (FitResid) for sample 1 and sample 2.

	Sample 1		Sample 2	
Item	Location	FitResid	Location	FitResid
**EX1**	-0.32	-1.63	-0.28	-1.11
**EX2**	-0.07	-3.49	-0.20	-0.77
**EX3**	-0.36	1.69	-0.47	0.76
**EX4**	-0.20	0.60	-0.31	-0.12
**EX5**	-0.63	1.68	-0.49	0.06
**EX6**	-0.06	-2.42	0.04	-3.88
**EX7**	-0.43	**4.41**	-0.47	2.18
**EX8**	-0.77	4.07	-0.89	3.35
**MD1**	0.07	-3.58	0.01	-0.42
**MD2**	-0.31	**4.84**	-0.20	**4.98**
**MD3**	0.31	-3.94	0.04	-1.50
**MD4**	0.23	1.23	0.16	2.31
**MD5**	-0.11	0.80	-0.30	3.39
**CI1**	-0.04	-0.89	0.05	-1.37
**CI2**	0.18	-4.73	0.47	-3.77
**CI3**	0.07	-0.21	0.29	1.20
**CI4**	0.06	-1.92	-0.05	-1.56
**CI5**	0.14	-1.00	0.25	0.73
**EI1**	0.71	-1.10	0.49	-2.77
**EI2**	0.55	-1.60	0.52	-3.06
**EI3**	-0.19	3.63	-0.14	**4.10**
**EI4**	0.74	-3.50	0.83	-2.68
**EI5**	0.43	3.62	0.66	0.72

Bold indicates significant item chi square (Bonferroni adj p-value <0.000435).

**Fig 3:**
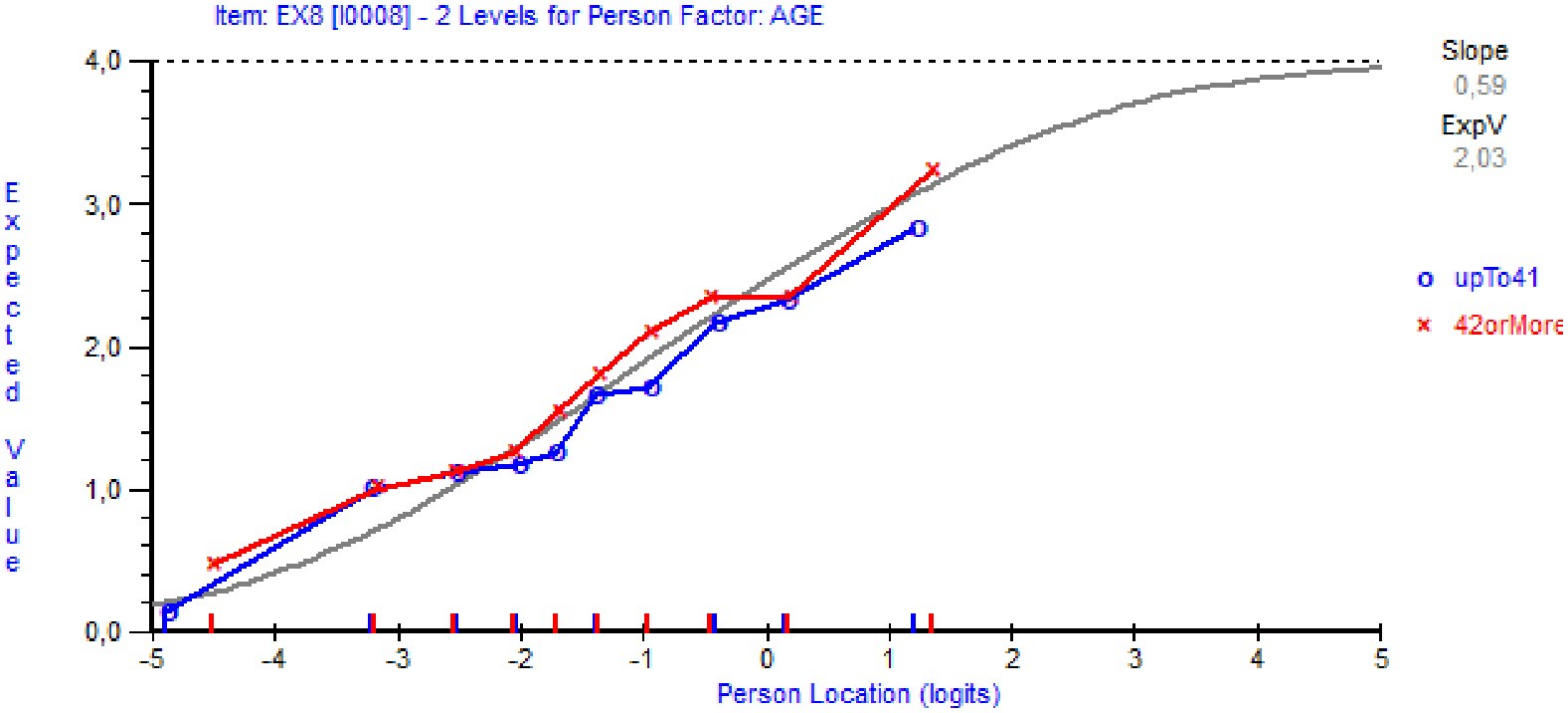
The item characteristic curve of item EX8, for older (over median age of 41) and younger (41 or younger) individuals.

Uniform DIF for age was noted for item EX8 (F_1,751_ = 17.11, p<0.0001). An example of the graphical evaluation of DIF is given in Fig 3. As seen in Fig 3, given the same level of burnout, older persons (above the median age of 41) score somewhat higher on this item compared to younger persons (41 years or younger). DIF for gender was observed for items EX8 (F_1,751_ = 18.51, p<0.0001, women scoring higher than men) and MD4 (F_1,751_ = 34.53; p<0.0001, men scoring higher), and for country items MD2 (F_1,751_ = 20.58, p<0.0001, NL scoring higher than FL), CI3 (F_1,751_ = 24.78, p<0.0001, FL scoring higher) and EI4 (F_1,751_ = 15.08; p<0.0001, NL scoring higher). Items CI2 (F_9,751_ = 3.81, p<0.0001) and EI4 (F_9,751_ = 4.59, p<0.0001) had problems with class intervals (misfit along the latent trait). At this stage of the analysis, no further investigation for DIF was done, as the focus was to first address issues with local dependency.

The next step was to fit the Rasch model on the four testlets, one for each subscale. The analysis of the four testlets resulted in a good fit to the model according to the summary fit statistics (Table 3, BAT 4 testlets). The item trait statistic was no longer significant. The result of Smith’s test was satisfying and showed that the percentage of significant t-tests dropped to 4.8 (3.5; 6.6). As expected, PSI decreased from 0.95 in the initial analysis to 0.85. The average latent correlation between the four testlets was 0.76 and when the four subscales are added together to make a total score, 92% of the total non-error variance was found to be common, which is further evidence that the responses on the four subscales can be summarized into a single score.

**Table 3:** Overall fit statistics in sample 1 and sample 2 (n = 800 each) and total sample of 2978.

	Item residual		Person residual		Chi square		Unidimensionality	
Analysis name	Mean	SD	Mean	SD	Value	p	PSI	Test % (95% CI)
**Sample 1 n = 800**								
BAT 23 items	-0.15	2.91	-0.86	2.86	416.51	<0.0001	0.95	20.9 (18.2;23.9)
BAT 4 testlets	0.07	1.17	-0.52	1.13	56.58	0.016	0.85	4.8 (3.5;6.6)
**Sample 2 n = 800**								
BAT 23 items	0.03	2.52	-0.77	2.41	378.55	<0.0001	0.95	22.8 (20.0;26.0)
BAT 4 testlets	0.22	1.73	-0.54	1.12	40.75	0.26	0.83	4.6 (3.3;6.3)
**Total sample n = 2978**								
BAT 23 items	-0.28	5.17	-0.80	2.48	970.30	<0.0001	0.95	21.1 (19.6;22.6)
BAT 4 testlets	-0.01	2.38	-0.53	1.1	109.65	<0.0001	0.85	4.4 (3.7;5.2)
***Ideal values***	***0*.*0***	***<1*.*4***	***0*.*0***	***<1*.*4***		***>0*.*01***	***>0*.*7***	***(LCI <5%)***

There was no DIF for age. DIF for gender was observed for the testlets exhaustion (F_1,751_ = 17.26, p<0.0001, women scored higher than men), mental distance (F_1,751_ = 37.34, p<0.0001, men scored higher), and DIF for country was observed for cognitive impairment (F_1,751_ = 19.46, p<0.0001, FL scored higher than NL). Next, DIF for gender was evaluated by splitting mental distance for gender, given that MD had the highest F-value. This resulted in the disappearance of gender DIF for the exhaustion testlet and thus indicated artificial DIF. This is also confirmed by the non-significant difference between the MD location values for women and men in the DIF resolved analysis (0.028 and -0.001 respectively, p-value 0.32). The differences between person mean values for women and men in the initial and the resolved analyses were 0.13 and 0.08 logits, respectively. The difference between CI locations between women and men was not significant in the DIF resolved analysis (0.07 and -0.094 respectively, p-values <0.0001). The difference between person mean values for women and men in the initial and resolved analyses were 0.13 and 0.11, respectively. Consequently, no adjustments for DIF were needed for either gender or country.

**Fig 4:**
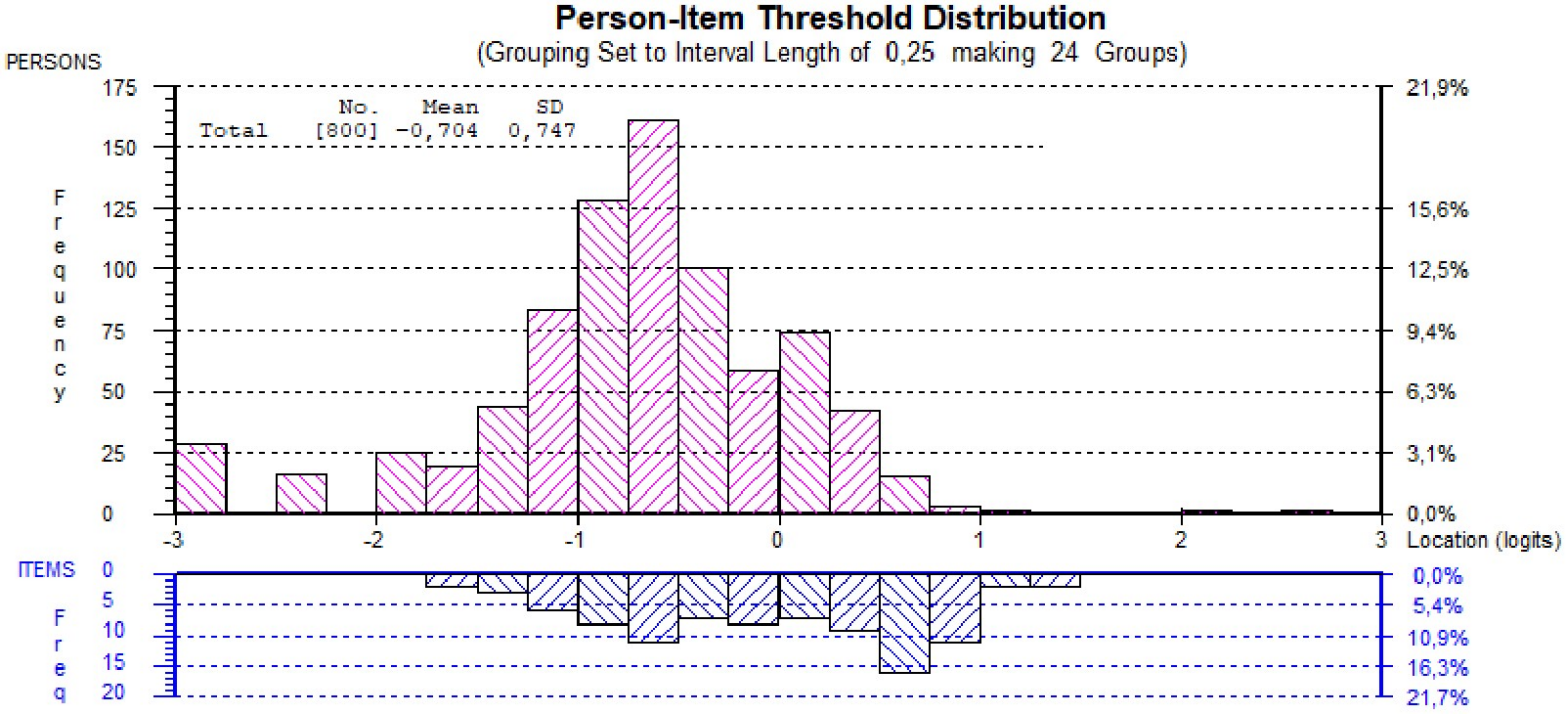
Person and item threshold distribution along the logit scale (higher values indicate higher burnout levels) using four testlets.

The distribution of item thresholds and study participants along the common logit scale (lower values indicate lower burnout levels) is shown in Fig 4. There is a group of participants with very low burnout levels (below -2 on a logit scale), which are lower levels of burnout than measured by the items. This is also illustrated by the person mean -0.704 (SD 0.747) compared to the item mean, which is constrained to 0, and further confirmed by the frequency distributions of each item. The highest response category (always) is rarely used, while approximately 50–75% of responses on each item endorsed the first two categories indicative of the lowest levels of burnout. Thus, the targeting was not optimal, but still acceptable.

### Rasch analysis using sample 2

All analyses were repeated on the second sample and the results were almost identical. Similar to sample 1, the residual correlation matrix from the initial analysis on the 23 items showed patterns that corresponded to the theoretical basis of BAT with four subscales (S2 Appendix in Table A2). The Smith’s test indicated a high percentage of significant t-tests (Table 3, sample 2. The fit to the Rasch model was not achieved according to the summary fit statistics in Table 3 –BAT 23 items).

All items had ordered thresholds. Items MD2 and EI3 had residuals outside the range of ±2.5 and a significant item chi-square. Non-significant residual scores, but outside the range, were observed for items EX6, EX8, MD5, CI2, EI1, EI2 and EI4 (Table 3). Items EX6 and EI3 had problems with class intervals in the DIF analysis (F_9,746_ = 3.87, p<0.0001 and F_9,746_ = 4.37, p<0.0001, respectively). There was no DIF for gender, whereas DIF for age was found for item CI2 (F_1,746_ = 15.20, p<0.0001; younger persons scored higher than older persons) and country CI3 (F_1,746_ = 23.36, p<0.0001, FL scored higher than NL).

Again, items were combined into four testlets based on the four BAT subscales and another Rasch analysis was performed. Model-fit was obtained, as shown in Table 3 (sample 2, BAT 4 testlets) which supported the hypothesis that the subscales could be added together into a single BAT score. The average latent correlation between the four testlets was 0.71 and the proportion of common non-error variances was 0.90.

DIF for age was noted for cognitive impairment, but additional analyses showed that there was no need for adjustment, as the difference between women’s and men’s location values for the CI testlet were not significant (0.007 and 0.047, respectively, p-value = 0.20). Differences in person mean values for women and in the initial and DIF resolved analyses were 0.13 and 0.26 logits, respectively. Targeting was similar as in sample 1 (figure not shown).

### Ordinal-to-interval conversion table

Given the fit to the Rasch model, ordinal scores (mean values of the 23 items) may be transformed into interval-level scores. Person scores obtained from the Rasch analysis are situated on a logit scale and can take both negative and positive values, with higher scores indicating higher levels of burnout. These logit scores are then linearly transformed into 1–5 interval scores, which is more intuitive and easier to interpret for BAT users. Table 4 provides interval scores in both logit units and in a 1–5 range, allowing users of the BAT to convert the ordinal mean score into interval-level (metric) scores. To increase precision, scores were calculated on the entire sample (n = 2978). Summary fit statistics for the total sample are shown in Table 3. The average latent correlation between the testlets was 0.74 and non-error common variance was 0.90. Conversion tables for the four subscales are given in S3 Appendix.

**Table 4:** Conversion table with raw mean scores on the Burnout Assessment Tool and their corresponding interval scale (metric) equivalents based on Rasch analysis (n = 2978).

Mean	Metric	Logit	Mean	Metric	Logit	Mean	Metric	Logit	Mean	Metric	Logit
1.00	1.00	-2.84	2.04	2.62	-0.64	3.09	3.15	0.08	4.13	3.63	0.73
1.04	1.40	-2.30	2.09	2.65	-0.61	3.13	3.17	0.11	4.17	3.65	0.76
1.09	1.64	-1.98	2.13	2.67	-0.58	3.17	3.19	0.14	4.22	3.67	0.79
1.13	1.78	-1.78	2.17	2.69	-0.55	3.22	3.21	0.17	4.26	3.69	0.82
1.17	1.89	-1.64	2.22	2.71	-0.52	3.26	3.24	0.20	4.30	3.71	0.84
1.22	1.97	-1.53	2.26	2.73	-0.49	3.30	3.26	0.23	4.35	3.73	0.87
1.26	2.03	-1.44	2.30	2.75	-0.46	3.35	3.28	0.26	4.39	3.75	0.91
1.30	2.09	-1.36	2.35	2.78	-0.43	3.39	3.30	0.28	4.43	3.78	0.94
1.35	2.14	-1.29	2.39	2.80	-0.40	3.43	3.32	0.31	4.48	3.81	0.97
1.39	2.18	-1.23	2.43	2.82	-0.37	3.48	3.34	0.34	4.52	3.83	1.01
1.43	2.23	-1.18	2.48	2.84	-0.34	3.52	3.36	0.37	4.57	3.86	1.05
1.48	2.26	-1.12	2.52	2.86	-0.31	3.57	3.38	0.39	4.61	3.90	1.10
1.52	2.30	-1.08	2.57	2.88	-0.28	3.61	3.40	0.42	4.65	3.93	1.15
1.57	2.33	-1.03	2.61	2.91	-0.25	3.65	3.42	0.45	4.70	3.98	1.21
1.61	2.37	-0.99	2.65	2.93	-0.22	3.70	3.44	0.47	4.74	4.02	1.27
1.65	2.40	-0.95	2.70	2.95	-0.19	3.74	3.46	0.50	4.78	4.08	1.35
1.70	2.42	-0.91	2.74	2.97	-0.16	3.78	3.47	0.52	4.83	4.15	1.45
1.74	2.45	-0.87	2.78	2.99	-0.13	3.83	3.49	0.55	4.87	4.25	1.57
1.78	2.48	-0.83	2.83	3.02	-0.10	3.87	3.51	0.57	4.91	4.38	1.76
1.83	2.50	-0.80	2.87	3.04	-0.07	3.91	3.53	0.60	4.96	4.61	2.01
1.87	2.53	-0.76	2.91	3.06	-0.04	3.96	3.55	0.63	5.00	5.00	2.60
1.91	2.55	-0.73	2.96	3.08	-0.01	4.00	3.57	0.65			
1.96	2.58	-0.70	3.00	3.11	0.02	4.04	3.59	0.68			
2.00	2.60	-0.67	3.04	3.13	0.05	4.09	3.61	0.70			

## Discussion

The aim of the current paper was threefold. Using Rasch analysis we evaluated: (a) the BAT’s construct validity consisting of four subscales; (b) whether the BAT’s four subscales could be combined to represent a single burnout score; and (c) whether differential item functioning regarding gender, age and country can be observed. Generally speaking, we have shown that the BAT has good psychometric properties after adjusting for local dependency between the items; i.e. when subscale scores instead of item scores are used. The BAT fulfils the criteria required by the Rasch measurement model and thus quantifies a latent trait of burnout. The first two aims were, therefore, achieved, as the results of the current study indicate that: a) the BAT consists of four subscales, and that b) these can be combined into a single burnout score. Moreover, each item works as intended regarding the ordering of the response categories. Finally, the BAT works invariantly for women and men, younger and older respondents, and across both the Netherlands and Flanders. That means that the third aim was also achieved. The mean scores of the BAT have been transformed into interval metric scores, which allows the use of parametric statistical techniques.

### A single burnout score

The residual correlation matrix in the initial analysis on the 23 items revealed that the items clustered within the BAT’s four subscales. The results of Smith’s test also indicated problems with violation of unidimensionality. This was not optimal from a measurement point of view. However, these results were not surprising because the clustering of the items was consistent with the underlying conceptualization of the BAT, consisting of four subscales, each representing a different aspect of burnout [20, 21]. Results like this are typically found for instruments consisting of bundles of items that measure different aspects of the latent dimension [39]. In fact, it illustrates that burnout is a syndrome consisting of four interrelated symptoms that all refer to one underlying deteriorated mental state. Our results with a strong general factor are also confirmed in a recent article that investigated the measurement invariance of the BAT across seven cross-national representative samples, and in which the BAT was modelled as a second-order factor and showed a good fit to the data [22]. In a similar vein, a recent study investigating the Japanese version of the BAT suggested the presence of a strong common factor as well [51].

Although the results make sense on theoretical grounds, it is nevertheless important to account for local dependency. Problems with local dependency influence the estimation of person parameters (metric scores) and inflate estimates of reliability (PSI), resulting in a false impression of the accuracy and precision of the estimates [38, 52]. We have accounted for local dependency by combining the correlated items into four testlets, which resulted in a good fit to the Rasch model. As expected, the PSI decreased from 0.95 in the initial analysis to 0.85 and 0.83 in the testlet analyses in both samples, respectively. The PSI in the total sample was 0.95 and 0.85 in the initial and testlet analyses respectively. However, the value of the PSI is still high enough to allow comparison of the BAT respondents with high precision. Moreover, the average latent correlation between the testlets and the percentage of common non-error variance was high and strengthens the conclusion that the responses of the four subscales can be summarized by a composite total burnout score. This is not possible with the MBI. In our study, the choice of particular items to form different testlets was straightforward, because the empirical evidence in the observed correlation patterns also indicated congruence with the definition of burnout, as measured by the BAT. Consequently, items were grouped according to the four subscales of the BAT: exhaustion, mental distance, and cognitive and emotional impairment. A solid theoretical rationale is a prerequisite to interpret the results of the Rasch analysis and is also emphasized in the psychometric literature. For instance, Rosenbaum states that the content of psychological tests should be guided by empirical evidence, but should not be mechanically determined by the outcome of statistical tests [53].

The MBI has been criticized for skewed answering patterns that may affect its reliability [15] and also for having disordered thresholds [32, 33]. All BAT items had ordered thresholds.

The estimates of the item thresholds need to be ordered, as they are partitioning the latent continuum (of burnout) into ordered categories. This property of monotonicity is a basic psychometric prerequisite, which is, however, often assumed only implicitly. An advantage of the Rasch analysis is that this requirement is formally tested. This means that respondents are using the item response categories (never to always) as intended by the developers. The ordering of the categories should be consistent with the person’s burnout level. A lower category should correspond to a lower level of burnout, whereas a higher category should correspond to a higher level of burnout. The increasing level of burnout severity across the categories was indeed reflected in the data and this was true for all 23 BAT items.

### Differential item functioning

The evaluation of DIF is important for any instrument that is to be used in different groups. The idea of invariant measures has already been mentioned by Thurstone [54]. If the frame of reference contains men and women, younger and older respondents, and participants from different countries, it is assumed that the model and the set of item parameters are similar for all comparable groups. Problems with DIF can be resolved by either splitting the DIF item for different groups or by deleting the DIF item from the scale [49]. In both scenarios there are effects on construct validity. Splitting the item for DIF may improve the fit, but the relative location estimates of the DIF items that are resolved are no longer invariant across the groups. An alternative option is to delete the DIF item from the instrument, but this affects the precision of the instrument. Each item is selected given its relevance, and removing an item implies losing information about the aspect of burnout which the developers considered as important. Therefore, before deciding what to do with a DIF item, it is crucial to evaluate whether it is a real or artificial DIF [49]. The results with the BAT indicated DIF for gender for exhaustion and mental distance testlets, and DIF for country regarding cognitive impairment in the first sample, and DIF for age and cognitive impairment in the second sample. However, additional analyses confirmed that this was artificial rather than real DIF. Consequently, no adjustments were needed. This essentially means that the BAT can be used in a similar way for men and women, younger and older respondents, and employees from the Netherlands and Flanders.

### Targeting

The targeting in the current study was acceptable. The mean location of the persons on the logit scale was lower than the predefined value of zero for the items, which is what one would expect to find if the scale functions as intended, given the fact that this is a representative sample of the working population and hence basically includes healthy persons. Given the good fit to the model, an ordinal-to-interval conversion table was presented. This is possible since the total score from the Rasch analysis is a sufficient statistic for estimating a person’s level of burnout, given that the data fit the Rasch measurement model. We recommend the use of metric values instead of mean scores to obtain better precision. An essential feature of any measurement implies equal intervals across the entire continuum of the construct being measured, an assumption that is not valid for the mean scores. The increase of one unit does not imply the same magnitude of burnout along the entire burnout continuum. This problem might not be that serious in the middle of the scale but is more pronounced toward both ends of the scale. Interestingly, it is toward the upper end of the scale that we would expect to find persons at risk of burnout. This problem is not in any way unique for the BAT; instead, it is a well-known fact that is true for many scales based on ordinal data [55, 56].

### Practical implications

For the users of the BAT we recommend first calculating the mean score for each person based on the item coding 1 to 5 (never to always). Then use the conversion table to translate each person’s mean score into the corresponding metric value. In this way a new variable can be created which will measure burnout on an interval level. Thus, using the conversion table allows for increased precision of the burnout scale. This new variable should be used in further analyses, e.g. for calculation of population average burnout levels and accompanied standard deviations. The conversion table is valid only for complete answers on all BAT items (no missing values are allowed). Moreover, metric scores for each of the four BAT subscales are also presented. These scores can be used to further differentiate the picture, which is particularly important for individual burnout assessment.

The BAT can be used as a screening device in organizations to identify employees who are at risk of burnout (i.e. have high or very high scores). For the interpretation of the BAT scores in terms of high and low burnout, we recommend consulting the BAT test manual, where the statistical norms for the Netherlands and Flanders (Belgium) are presented [21]. Statistical norms are based on percentiles and classify population into four categories: low, average, high and very high. These statistical norms make it possible to assess the level of burnout of individuals and groups, based on a comparison with the “average” Flemish or Dutch employee. The use of statistical norms based on national representative samples are clearly an advantage of the BAT over the MBI. Another advantage is that the BAT does not include reversed items, whereas the MBI has been criticized for including positively worded items in the professional efficacy scale [16]. A direct comparison of the BAT and the MBI was out of the scope for this study. However, such a comparison would be interesting and relevant in future studies.

### Strengths and weaknesses

An advantage of the current study is that the data come from large, representative samples of the working population in the Netherlands and Flanders (Belgium). On the other hand, large samples could also be a disadvantage, because even minor levels of misfit become statistically significant when chi-square statistics are used. To overcome this problem, two random samples were selected with 800 participants each, which were still large enough to satisfy the recommended sample size for performing a Rasch analysis. In addition, this makes it possible to cross-validate the results. If there were any major problems with the scale, these should have emerged in both subsamples. The analyses were done using the same Dutch language version, so that further validation of other language versions of the BAT still stands out. In this study, validation was carried out on a sample from two working populations. Further studies should also focus on the validation of the BAT in patients with (severe) burnout.

Lastly, this is the first time the BAT was evaluated thoroughly using the Rasch measurement model. Usually, the goal of many statistical analyses is to fit the model that best describes the data. The opposite is true when fitting the data to the Rasch model. The Rasch analysis tests whether the *data* fits the requirements of the Rasch model. From a psychometric point of view, the scale shows criterion-related construct validity if the requirements of unidimensionality, monotonicity, invariance, DIF and local dependency are met [53]. Our results show that all these requirements are met, because the data fit the Rasch model [26]. In other words, we can conclude that the BAT is a construct-valid measurement of burnout.

## Conclusion

Using data from representative samples of working populations in the Netherlands and Flanders (Belgium), this study demonstrated that the newly developed BAT (Burnout Assessment Tool) has sound psychometric properties and fulfils the measurement criteria according to the Rasch model. The BAT score reflects the scoring structure indicated by the developers of the scale and makes it possible to summarize the level of burnout into a single burnout score. The BAT score also works invariantly for women and men, younger and older respondents, and across both countries. Hence, the BAT can be used in organizations for screening and identifying employees who are at risk of burnout.

## Supporting information

S1 Appendix The Burnout Assessment Tool (BAT). (PDF)

S2 Appendix The observed residual correlation matrix for the Burnout Assessment Tool. (DOCX)

S3 Appendix Conversion tables from mean into metric score for the four BAT subscales. (DOCX)

S1 Data Complete data n = 2978. (XLSX)

S2 Data Sample 1 n = 800. (XLSX)

S3 Data Sample 2 n = 800. (XLSX)
