# Supplementary material for: A Rasch analysis of the Burnout Assessment Tool (BAT)
Source: PLoS One. 2020 Nov 24;15(11):e0242241. doi: 10.1371/journal.pone.0242241 (PMC7685472; doi:10.1371/journal.pone.0242241)
Supplement: S1 Appendix — (PDF) [file pone.0242241.s001.pdf]

# Work-related version of the BAT

*Instruction*

The following statements are related to your work situation and how you experience this situation. Please state how often each statement applies to you.

*Scoring*

| Never | Rarely | Sometimes | Often | Always |
|---|---|---|---|---|
| 1 | 2 | 3 | 4 | 5 |

*Questions*

| | Never | Rarely | Sometimes | Often | Always |
|---|---|---|---|---|---|
| ***Exhaustion*** | | | | | |
| At work, I feel mentally exhausted | ☐ | ☐ | ☐ | ☐ | ☐ |
| Everything I do at work requires a great deal of effort | ☐ | ☐ | ☐ | ☐ | ☐ |
| After a day at work, I find it hard to recover my energy | ☐ | ☐ | ☐ | ☐ | ☐ |
| At work, I feel physically exhausted | ☐ | ☐ | ☐ | ☐ | ☐ |
| When I get up in the morning, I lack the energy to start a new day at work | ☐ | ☐ | ☐ | ☐ | ☐ |
| I want to be active at work, but somehow I am unable to manage | ☐ | ☐ | ☐ | ☐ | ☐ |
| When I exert myself at work, I quickly get tired | ☐ | ☐ | ☐ | ☐ | ☐ |
| At the end of my working day, I feel mentally exhausted and drained | ☐ | ☐ | ☐ | ☐ | ☐ |
| ***Mental distance*** | | | | | |
| I struggle to find any enthusiasm for my work | ☐ | ☐ | ☐ | ☐ | ☐ |
| At work, I do not think much about what I am doing and I function on autopilot | ☐ | ☐ | ☐ | ☐ | ☐ |
| I feel a strong aversion towards my job | ☐ | ☐ | ☐ | ☐ | ☐ |
| I feel indifferent about my job | ☐ | ☐ | ☐ | ☐ | ☐ |
| I'm cynical about what my work means to others | ☐ | ☐ | ☐ | ☐ | ☐ |
| ***Cognitive impairment*** | | | | | |
| At work, I have trouble staying focused | ☐ | ☐ | ☐ | ☐ | ☐ |
| At work I struggle to think clearly | ☐ | ☐ | ☐ | ☐ | ☐ |
| I'm forgetful and distracted at work | ☐ | ☐ | ☐ | ☐ | ☐ |
| When I'm working, I have trouble concentrating | ☐ | ☐ | ☐ | ☐ | ☐ |
| I make mistakes in my work because I have my mind on other things | ☐ | ☐ | ☐ | ☐ | ☐ |
| ***Emotional impairment*** | | | | | |
| At work, I feel unable to control my emotions | ☐ | ☐ | ☐ | ☐ | ☐ |
| I do not recognize myself in the way I react emotionally at work | ☐ | ☐ | ☐ | ☐ | ☐ |

Citation: Schaufeli, W.B., De Witte, H. & Desart, S. (2019). *Handleiding Burnout Assessment Tool (BAT).* KU Leuven, België: Intern rapport.
*With the help of Russell Watts – Operations Director, Publishing and Ventures @ Penguin Random House UK*

| | | | | | |
|---|---|---|---|---|---|
| During my work I become irritable when things don't go my way | ☐ | ☐ | ☐ | ☐ | ☐ |
| I get upset or sad at work without knowing why | ☐ | ☐ | ☐ | ☐ | ☐ |
| At work I may overreact unintentionally | ☐ | ☐ | ☐ | ☐ | ☐ |

| | Never | Rarely | Sometimes | Often | Always |
|---|---|---|---|---|---|
| ***Psychological complaints*** | | | | | |
| I have trouble falling or staying asleep | ☐ | ☐ | ☐ | ☐ | ☐ |
| I tend to worry | ☐ | ☐ | ☐ | ☐ | ☐ |
| I feel tense and stressed | ☐ | ☐ | ☐ | ☐ | ☐ |
| I feel anxious and/or suffer from panic attacks | ☐ | ☐ | ☐ | ☐ | ☐ |
| Noise and crowds disturb me | ☐ | ☐ | ☐ | ☐ | ☐ |
| ***Psychosomatic complaints*** | | | | | |
| I suffer from palpitations or chest pain | ☐ | ☐ | ☐ | ☐ | ☐ |
| I suffer from stomach and/or intestinal complaints | ☐ | ☐ | ☐ | ☐ | ☐ |
| I suffer from headaches | ☐ | ☐ | ☐ | ☐ | ☐ |
| I suffer from muscle pain, for example in the neck, shoulder or back | ☐ | ☐ | ☐ | ☐ | ☐ |
| I often get sick | ☐ | ☐ | ☐ | ☐ | ☐ |

Citation: Schaufeli, W.B., De Witte, H. & Desart, S. (2019). *Handleiding Burnout Assessment Tool (BAT).* KU Leuven, België: Intern rapport.
*With the help of Russell Watts – Operations Director, Publishing and Ventures @ Penguin Random House UK*
