# Supplementary material for: A Rasch analysis of the Burnout Assessment Tool (BAT)
Source: PLoS One. 2020 Nov 24;15(11):e0242241. doi: 10.1371/journal.pone.0242241 (PMC7685472; doi:10.1371/journal.pone.0242241)
Supplement: S2 Appendix — (DOCX) [file pone.0242241.s002.docx]

**S2 Appendix.** Table A1. The observed residual correlation matrix for the Burnout Inventory Tool. Representative sample of working population NL and FL, subsample 1 n=800.

| **Item** | **EX1** | **EX2** | **EX3** | **EX4** | **EX5** | **EX6** | **EX7** | **EX8** | **MD1** | **MD2** | **MD3** | **MD4** | **MD5** | **CI1** | **CI2** | **CI3** | **CI4** | **CI5** | **EI1** | **EI2** | **EI3** | **EI4** | **EI5** |
| --- | --- | --- | --- | --- | --- | --- | --- | --- | --- | --- | --- | --- | --- | --- | --- | --- | --- | --- | --- | --- | --- | --- | --- |
| **EX1** | 1.00 |  |  |  |  |  |  |  |  |  |  |  |  |  |  |  |  |  |  |  |  |  |  |
| **EX2** | .06 | 1.00 |  |  |  |  |  |  |  |  |  |  |  |  |  |  |  |  |  |  |  |  |  |
| **EX3** | .15 | .07 | 1.00 |  |  |  |  |  |  |  |  |  |  |  |  |  |  |  |  |  |  |  |  |
| **EX4** | .21 | .07 | .17 | 1.00 |  |  |  |  |  |  |  |  |  |  |  |  |  |  |  |  |  |  |  |
| **EX5** | .02 | .13 | .21 | .07 | 1.00 |  |  |  |  |  |  |  |  |  |  |  |  |  |  |  |  |  |  |
| **EX6** | .03 | .04 | .04 | .09 | .07 | 1.00 |  |  |  |  |  |  |  |  |  |  |  |  |  |  |  |  |  |
| **EX7** | .08 | .05 | .12 | .18 | .00 | .05 | 1.00 |  |  |  |  |  |  |  |  |  |  |  |  |  |  |  |  |
| **EX8** | .30 | .05 | .28 | .27 | .10 | -.04 | .18 | 1.00 |  |  |  |  |  |  |  |  |  |  |  |  |  |  |  |
| **MD1** | -.08 | -.01 | -.15 | -.11 | -.01 | .04 | -.21 | -.16 | 1.00 |  |  |  |  |  |  |  |  |  |  |  |  |  |  |
| **MD2** | -.11 | -.09 | -.14 | -.04 | -.06 | -.11 | -.16 | -.06 | .14 | 1.00 |  |  |  |  |  |  |  |  |  |  |  |  |  |
| **MD3** | .00 | .06 | -.09 | -.09 | -.02 | -.02 | -.15 | -.11 | .30 | .17 | 1.00 |  |  |  |  |  |  |  |  |  |  |  |  |
| **MD4** | -.18 | .00 | -.21 | -.18 | -.08 | -.05 | -.18 | -.19 | .35 | .23 | .30 | 1.00 |  |  |  |  |  |  |  |  |  |  |  |
| **MD5** | -.10 | -.04 | -.06 | -.14 | -.04 | -.07 | -.15 | -.11 | .24 | .19 | .14 | .27 | 1.00 |  |  |  |  |  |  |  |  |  |  |
| **CI1** | -.13 | -.10 | -.21 | -.18 | -.12 | -.08 | -.04 | -.20 | -.04 | -.11 | -.12 | -.08 | -.14 | 1.00 |  |  |  |  |  |  |  |  |  |
| **CI2** | .01 | -.11 | -.23 | -.13 | -.12 | -.07 | -.12 | -.13 | -.16 | -.15 | -.15 | -.19 | -.16 | .32 | 1.00 |  |  |  |  |  |  |  |  |
| **CI3** | -.16 | -.10 | -.19 | -.15 | -.14 | -.01 | -.08 | -.24 | -.11 | -.14 | -.19 | -.08 | -.13 | .33 | .34 | 1.00 |  |  |  |  |  |  |  |
| **CI4** | -.13 | -.09 | -.24 | -.16 | -.10 | -.03 | -.11 | -.21 | -.11 | -.10 | -.17 | -.09 | -.13 | .44 | .46 | .34 | 1.00 |  |  |  |  |  |  |
| **CI5** | -.21 | -.16 | -.19 | -.23 | -.16 | -.08 | .00 | -.22 | -.14 | -.13 | -.17 | -.08 | -.19 | .23 | .27 | .35 | .28 | 1.00 |  |  |  |  |  |
| **EI1** | -.15 | -.11 | -.10 | -.21 | -.17 | -.14 | -.14 | -.16 | -.16 | -.16 | -.12 | -.16 | -.11 | -.06 | -.03 | -.10 | -.06 | -.01 | 1.00 |  |  |  |  |
| **EI2** | -.16 | -.14 | -.15 | -.17 | -.27 | -.13 | -.15 | -.18 | -.16 | -.13 | -.08 | -.06 | -.12 | -.09 | -.06 | -.03 | -.09 | .05 | .41 | 1.00 |  |  |  |
| **EI3** | -.14 | -.15 | -.08 | -.16 | -.12 | -.18 | -.14 | -.20 | -.13 | -.10 | -.19 | -.11 | -.04 | -.10 | -.05 | -.03 | -.07 | .04 | .19 | .16 | 1.00 |  |  |
| **EI4** | -.14 | -.13 | -.10 | -.14 | -.19 | -.10 | -.18 | -.18 | -.12 | -.13 | -.07 | -.12 | -.18 | -.09 | -.02 | -.11 | -.10 | .03 | .40 | .42 | .16 | 1.00 |  |
| **EI5** | -.18 | -.19 | -.13 | -.19 | -.21 | -.15 | -.14 | -.19 | -.15 | -.13 | -.16 | -.14 | -.11 | -.11 | -.01 | -.06 | -.10 | .00 | .39 | .33 | .34 | .33 | 1.00 |

Table A2. The observed residual correlation matrix for the Burnout Inventory Tool. Representative sample of working population NL and FL, subsample 2 n=800.

| **Item** | **EX1** | **EX2** | **EX3** | **EX4** | **EX5** | **EX6** | **EX7** | **EX8** | **MD1** | **MD2** | **MD3** | **MD4** | **MD5** | **CI1** | **CI2** | **CI3** | **CI4** | **CI5** | **EI1** | **EI2** | **EI3** | **EI4** | **EI5** |
| --- | --- | --- | --- | --- | --- | --- | --- | --- | --- | --- | --- | --- | --- | --- | --- | --- | --- | --- | --- | --- | --- | --- | --- |
| **EX1** | 1.00 |  |  |  |  |  |  |  |  |  |  |  |  |  |  |  |  |  |  |  |  |  |  |
| **EX2** | .06 | 1.00 |  |  |  |  |  |  |  |  |  |  |  |  |  |  |  |  |  |  |  |  |  |
| **EX3** | .12 | .10 | 1.00 |  |  |  |  |  |  |  |  |  |  |  |  |  |  |  |  |  |  |  |  |
| **EX4** | .17 | .20 | .30 | 1.00 |  |  |  |  |  |  |  |  |  |  |  |  |  |  |  |  |  |  |  |
| **EX5** | .04 | .08 | .19 | .09 | 1.00 |  |  |  |  |  |  |  |  |  |  |  |  |  |  |  |  |  |  |
| **EX6** | .10 | .12 | .06 | .06 | .19 | 1.00 |  |  |  |  |  |  |  |  |  |  |  |  |  |  |  |  |  |
| **EX7** | .15 | .13 | .21 | .28 | .03 | .00 | 1.00 |  |  |  |  |  |  |  |  |  |  |  |  |  |  |  |  |
| **EX8** | .26 | .04 | .26 | .22 | .05 | .07 | .15 | 1.00 |  |  |  |  |  |  |  |  |  |  |  |  |  |  |  |
| **MD1** | -.11 | -.03 | -.20 | -.13 | -.03 | -.02 | -.24 | -.22 | 1.00 |  |  |  |  |  |  |  |  |  |  |  |  |  |  |
| **MD2** | -.12 | -.12 | -.12 | -.12 | -.04 | -.03 | -.09 | -.15 | .29 | 1.00 |  |  |  |  |  |  |  |  |  |  |  |  |  |
| **MD3** | -.02 | .04 | -.14 | -.08 | .03 | -.07 | -.21 | -.16 | .48 | .17 | 1.00 |  |  |  |  |  |  |  |  |  |  |  |  |
| **MD4** | -.17 | -.05 | -.20 | -.21 | -.04 | -.05 | -.23 | -.24 | .50 | .41 | .43 | 1.00 |  |  |  |  |  |  |  |  |  |  |  |
| **MD5** | -.12 | -.09 | -.18 | -.19 | -.08 | -.13 | -.15 | -.18 | .36 | .20 | .30 | .36 | 1.00 |  |  |  |  |  |  |  |  |  |  |
| **CI1** | -.12 | -.08 | -.12 | -.21 | -.09 | -.06 | -.11 | -.10 | -.18 | -.13 | -.19 | -.17 | -.16 | 1.00 |  |  |  |  |  |  |  |  |  |
| **CI2** | -.12 | -.12 | -.07 | -.16 | -.14 | -.08 | -.07 | -.14 | -.23 | -.22 | -.28 | -.28 | -.24 | .40 | 1.00 |  |  |  |  |  |  |  |  |
| **CI3** | -.15 | -.21 | -.15 | -.20 | -.12 | -.12 | -.15 | -.09 | -.20 | -.19 | -.26 | -.19 | -.16 | .28 | .37 | 1.00 |  |  |  |  |  |  |  |
| **CI4** | -.16 | -.14 | -.13 | -.24 | -.08 | .01 | -.15 | -.12 | -.17 | -.14 | -.23 | -.19 | -.18 | .48 | .43 | .34 | 1.00 |  |  |  |  |  |  |
| **CI5** | -.14 | -.16 | -.18 | -.21 | -.17 | -.14 | -.08 | -.08 | -.26 | -.17 | -.25 | -.23 | -.17 | .23 | .30 | .39 | .27 | 1.00 |  |  |  |  |  |
| **EI1** | -.13 | -.14 | -.18 | -.16 | -.28 | -.15 | -.14 | -.18 | -.19 | -.19 | -.15 | -.18 | -.12 | -.05 | .05 | .04 | -.01 | .09 | 1.00 |  |  |  |  |
| **EI2** | -.11 | -.12 | -.20 | -.15 | -.24 | -.14 | -.14 | -.18 | -.18 | -.14 | -.14 | -.15 | -.12 | -.05 | .01 | -.01 | -.04 | .08 | .44 | 1.00 |  |  |  |
| **EI3** | -.19 | -.19 | -.18 | -.10 | -.15 | -.17 | -.10 | -.14 | -.14 | -.12 | -.15 | -.17 | -.02 | -.09 | .02 | .04 | -.01 | .06 | .20 | .18 | 1.00 |  |  |
| **EI4** | -.17 | -.12 | -.14 | -.20 | -.18 | -.17 | -.15 | -.17 | -.11 | -.16 | -.07 | -.09 | -.11 | -.06 | -.02 | -.02 | -.09 | .01 | .45 | .42 | .14 | 1.00 |  |
| **EI5** | -.11 | -.19 | -.18 | -.13 | -.20 | -.17 | -.15 | -.16 | -.20 | -.21 | -.21 | -.18 | -.12 | -.10 | .03 | .03 | -.07 | .07 | .43 | .39 | .33 | .42 | 1.00 |
