# Supplementary material for: A Rasch analysis of the Burnout Assessment Tool (BAT)
Source: PLoS One. 2020 Nov 24;15(11):e0242241. doi: 10.1371/journal.pone.0242241 (PMC7685472; doi:10.1371/journal.pone.0242241)
Supplement: S3 Appendix — (DOCX) [file pone.0242241.s003.docx]

## S3 Appendix

Table 1. Conversion table with raw mean scores on the Exhaustion subscale of the Burnout Assessment Tool and their corresponding interval scale (metric and logit) equivalents based on Rasch analysis (n=2978).

| **Mean** | **Metric** | **Logit** |  | **Mean** | **Metric** | **Logit** |  |
| --- | --- | --- | --- | --- | --- | --- | --- |
| 1.00 | 1.00 | -2.05 |  | 3.13 | 3.32 | -0.02 |  |
| 1.13 | 1.36 | -1.74 |  | 3.25 | 3.42 | 0.06 |  |
| 1.25 | 1.61 | -1.52 |  | 3.38 | 3.51 | 0.14 |  |
| 1.38 | 1.79 | -1.36 |  | 3.50 | 3.60 | 0.22 |  |
| 1.50 | 1.93 | -1.24 |  | 3.63 | 3.69 | 0.30 |  |
| 1.63 | 2.06 | -1.13 |  | 3.75 | 3.77 | 0.37 |  |
| 1.75 | 2.17 | -1.03 |  | 3.88 | 3.85 | 0.44 |  |
| 1.88 | 2.29 | -0.93 |  | 4.00 | 3.93 | 0.51 |  |
| 2.00 | 2.40 | -0.83 |  | 4.13 | 4.01 | 0.58 |  |
| 2.13 | 2.51 | -0.74 |  | 4.25 | 4.10 | 0.65 |  |
| 2.25 | 2.61 | -0.64 |  | 4.38 | 4.18 | 0.73 |  |
| 2.38 | 2.72 | -0.55 |  | 4.50 | 4.28 | 0.81 |  |
| 2.50 | 2.83 | -0.46 |  | 4.63 | 4.38 | 0.90 |  |
| 2.63 | 2.93 | -0.37 |  | 4.75 | 4.52 | 1.02 |  |
| 2.75 | 3.03 | -0.28 |  | 4.88 | 4.71 | 1.19 |  |
| 2.88 | 3.13 | -0.19 |  | 5.00 | 5.00 | 1.44 |  |
| 3.00 | 3.23 | -0.10 |  |  |  |  |  |

Table 2. Conversion table with raw mean scores on the Mental distance subscale of the Burnout Assessment Tool and their corresponding interval scale (metric and logit) equivalents based on Rasch analysis (n=2978).

| **Mean** | **Metric** | **Logit** |
| --- | --- | --- |
| 1.00 | 1.00 | -1.24 |
| 1.20 | 1.32 | -1.05 |
| 1.40 | 1.57 | -0.89 |
| 1.60 | 1.76 | -0.78 |
| 1.80 | 1.92 | -0.68 |
| 2.00 | 2.07 | -0.59 |
| 2.20 | 2.23 | -0.49 |
| 2.40 | 2.40 | -0.39 |
| 2.60 | 2.59 | -0.27 |
| 2.80 | 2.81 | -0.14 |
| 3.00 | 3.05 | 0.01 |
| 3.20 | 3.29 | 0.16 |
| 3.40 | 3.50 | 0.28 |
| 3.60 | 3.68 | 0.39 |
| 3.80 | 3.84 | 0.49 |
| 4.00 | 3.98 | 0.57 |
| 4.20 | 4.13 | 0.66 |
| 4.40 | 4.28 | 0.76 |
| 4.60 | 4.46 | 0.86 |
| 4.80 | 4.69 | 1.00 |
| 5.00 | 5.00 | 1.19 |

Table 3. Conversion table with raw mean scores on the Cognitive impairment subscale of the Burnout Assessment Tool and their corresponding interval scale (metric and logit) equivalents based on Rasch analysis (n=2978).

| **Mean** | **Metric** | **Logit** |
| --- | --- | --- |
| 1.00 | 1.00 | -1.75 |
| 1.20 | 1.42 | -1.43 |
| 1.40 | 1.74 | -1.19 |
| 1.60 | 1.98 | -1.01 |
| 1.80 | 2.19 | -0.85 |
| 2.00 | 2.40 | -0.69 |
| 2.20 | 2.61 | -0.54 |
| 2.40 | 2.83 | -0.37 |
| 2.60 | 3.07 | -0.19 |
| 2.80 | 3.31 | 0.00 |
| 3.00 | 3.55 | 0.17 |
| 3.20 | 3.75 | 0.33 |
| 3.40 | 3.92 | 0.45 |
| 3.60 | 4.05 | 0.55 |
| 3.80 | 4.17 | 0.64 |
| 4.00 | 4.28 | 0.73 |
| 4.20 | 4.37 | 0.80 |
| 4.40 | 4.49 | 0.89 |
| 4.60 | 4.62 | 0.98 |
| 4.80 | 4.79 | 1.11 |
| 5.00 | 5.00 | 1.27 |

Table 4. Conversion table with raw mean scores on the Emotional impairment subscale of the Burnout Assessment Tool and their corresponding interval scale (metric and logit) equivalents based on Rasch analysis (n=2978).

| **Mean** | **Metric** | **Logit** |
| --- | --- | --- |
| 1.00 | 1.00 | -1.22 |
| 1.20 | 1.33 | -0.98 |
| 1.40 | 1.57 | -0.80 |
| 1.60 | 1.75 | -0.67 |
| 1.80 | 1.91 | -0.56 |
| 2.00 | 2.05 | -0.45 |
| 2.20 | 2.19 | -0.35 |
| 2.40 | 2.34 | -0.24 |
| 2.60 | 2.50 | -0.12 |
| 2.80 | 2.68 | 0.01 |
| 3.00 | 2.88 | 0.15 |
| 3.20 | 3.08 | 0.30 |
| 3.40 | 3.27 | 0.44 |
| 3.60 | 3.46 | 0.57 |
| 3.80 | 3.63 | 0.70 |
| 4.00 | 3.80 | 0.82 |
| 4.20 | 3.96 | 0.94 |
| 4.40 | 4.14 | 1.07 |
| 4.60 | 4.34 | 1.22 |
| 4.80 | 4.62 | 1.42 |
| 5.00 | 5.00 | 1.70 |
